# Identification of etiological agents by LPA and PCR in childhood meningitis

**DOI:** 10.12669/pjms.295.3902

**Published:** 2013

**Authors:** Mashal Khan, Khalid Mahmood A. Khan, Khatidja Pardhan, Ashfaque Ahmed Memon

**Affiliations:** 1 Mashal Khan, MBBS, DCH, MCPS, FCPS, Assistant Professor, Medical Unit, National Institute of Child Health (NICH), Karachi, Pakistan.; 2 Khalid Mahmood A. Khan, MBBS, MCPS, FCPS, Associate Professor, Medical Unit, National Institute of Child Health (NICH), Karachi, Pakistan.; 3 Dr. Khatidja Pardhan, MBBS, FCPS-II, Medical Officer, Medical Unit, National Institute of Child Health (NICH), Karachi, Pakistan.; 4 Mr. Ashfaque Ahmed Memon, MSC, Statistical Officer PMRC, National Institute of Child Health (NICH), Karachi, Pakistan.

**Keywords:** Etiological Agents, Meningitis

## Abstract

***Objectives: ***To determine the etiological agents by Latex Particle Agglutination (LPA) and Polymerase Chain Reaction (PCR) in patients admitted with Cerebrospinal Fluid (CSF) culture negative bacterial meningitis

***Methods: ***This descriptive case series was conducted at National Institute of Child Health, Karachi from January 2010 to December 2012. Patients meeting the WHO case definition of suspected meningitis from one month to 59 months of age were included in the study. CSF examination and culture was carried out on every patient and CSF culture negative patients were enrolled. Demographic data, clinical signs & symptoms and laboratory findings were entered into the proforma. Data was analyzed using statistical package for social sciences (SPSS) version 17. P-value <0.05 was taken as significant.

***Results: ***A total of 166 patients were included. Male were 96 and female were 76 with the male to female ratio of 1.26. The mean age of patient was ± SD 14.6 ± 14.5 months. The etiological agents identified by LPA were in 26/166 (15.66%) cases and the organisms were H. influenzae type b 10 cases, streptococcus pneumoniae 15 cases and meningococcus only one case respectively. The organisms identified by PCR were in 65/166 (39.15%) cases and the isolates were H. influenzae type b 16 cases, streptococcus pneumoniae 48 cases and meningococcus 01 case respectively.

***Conclusion: ***LPA and PCR are superior and useful diagnostic tools in microbiology. They can be used for rapid etiological diagnosis of bacterial meningitis for the early administration of proper antibiotic.

***Abbreviation:*** LPA = Latex Particle Agglutination, PCR = Polymerase Chain Reaction, CSF = Cerebrospinal Fluid, CNS = Central Nervous System.

## INTRODUCTION

Bacterial meningitis is a serious form of meningo-encephalitis that causes inflammation of meninges of the brain, particularly the arachnoid and piamater.^1^ Apart from meningeal inflammation, it has also shown to affect other regions of the central nervous system (CNS). It is notorious for producing detrimental long-term clinical manifestations and life threatening consequences in comparison to aseptic meningitis.^1^^,^^2^

Bacterial Meningitis is a medical emergency and immediate diagnostic steps must be taken to establish the specific cause so that appropriate antimicrobial therapy can be initiated. The mortality rate of untreated bacterial meningitis approaches 100%, even with optimal therapy, mortality and morbidity may occur.^3^

The common pathogens causing bacterial meningitis after neonatal period are haemophilus influenzae type b, (Hib) streptococcus pneumoniae (Sp) and meningococcus. S. pneumonia and meningococcus contribute to 61% of cases of meningitis.^2^^,^^4^ Hib meningitis is a disease affecting primarily young children; most of the cases occur in children one month to 3 years of age.^5^ The use of Hib conjugate vaccine has reduced the incidence of, or has even virtually eliminated invasive Hib disease in some industrialized countries.^6^ Sp is a major cause of childhood bacterial meningitis in countries where Hib disease has been eliminated by vaccination. It is the second most frequently reported cause of septic meningitis in some European and sub-Saharan African countries, after meningococcal cases.^7^ Meningococcus is now considered to be the leading cause of meningitis in many regions of the world causing an estimated 1.2 million cases of bacterial meningitis and sepsis worldwide each year.^8^

Early etiological diagnosis and appropriate treatment of meningitis cases result in a higher cure rate and lower incidence of potentially fatal complications. The detection of bacterial antigens in cerebrospinal fluid (CSF) could be an important diagnostic tool for early empirical antibiotic therapy in bacterial meningitis.^9^

Latex Particle Agglutination (LPA) is a very useful tool in the diagnosis of bacterial meningitis with sensitivity and specificity ranging from 95-100%. It is easy to perform and interpret, require minimum time and the use of antibiotic generally do not alter the results when used for short interval.^10^

In the recent years, Polymerase chain reaction (PCR) based assays have become accessible and provide an early and accurate diagnosis of bacterial meningitis. PCR testing of CSF is more sensitive than CSF culture, particularly in patients who have received previous antimicrobials.^11^^,^^12^

The etiological diagnosis of bacterial meningitis in developing countries is difficult to establish for many reasons. Parents often administer antibiotic at the first sign of fever in their infants as these antibiotics are easily available from local markets. General Practitioners (GPs) also routinely prescribe antibiotics without doing blood C/S or any other tests. These antibiotics often result in negative CSF culture and alter CSF biochemistry in children with meningitis. Therefore, we undertook this study to assess the clinical efficacy of LPA and PCR in identifying etiological agents in patients when Gram staining and culture were negative.

## METHODS

This descriptive case series was conducted at National Institute of Child Health (NICH), Karachi from Jan 2010 to Dec 2012. Patients fulfilling the WHO case definition of meningitis i.e. sudden onset of fever >38.5c^0^ rectal or >38c^0 ^axillary and at least one of the following: headache, vomiting, neck-stiffness, bulging fontanelle, altered or reduced level of consciousness, convulsion, poor sucking and irritability were included in the study. Patients age ranged from 01 month to 59 months were recruited. Demographic data, clinical signs, symptoms and laboratory findings were assessed and recorded in the predesigned proforma. CBC, blood C/S, Gram stain, blood sugar, serum electrolyte and CSF examination was performed on every patient and CSF culture negative patients were enrolled in the study.

Neonates (<1month of age) were excluded because the pathogens causing neonatal meningitis are different from older children. Patients with tuberculous meningitis and acute viral meningitis were also excluded from the study. Implied consent was taken from parents/care giver. Approval was taken from the hospital ethical committee. Every patient was examined thoroughly. LPA and PCR techniques were performed on every patient to detect 03 specific antigens for Haemophlus influenzae type b, S. pneumoniae and meningococcus.

Patients were managed according to the standard protocol of hospital. Strict monitoring of vital signs of every patient was carried out. Patients were examined on regular basis for development of complications or progression of disease.

All the data was analyzed by using statistical package for social sciences (SPSS) version 17 . P-value <0.05 was taken significant.

## RESULTS

A total of 166 patients admitted with suspected bacterial meningitis during the defined study period were included in the study. Male were 96 and female were 76 with the male to female ratio was 1.26. The mean age of patients ± SD was 14.6 ± 14.55 months. Majority (77.5%) of patients had received antibiotic before admission. Patient from 6 months to 24 months were 138 and only 28 cases were from 24 to 59 months of age. Regarding the CSF examination, 15(9.03%) samples were found to be turbid on naked eye examination, 13(7.83%) samples were bloody and the rest of the samples had clear appearance. Twenty five (15%) samples had abnormal protein and glucose concentration. The CSF cell count in 19 samples of age group 1-24 months had 10-100 cells, 97 patient had 100-300 cell and 22 patients had numerous cells. In the age group from 25-59 months, only 02 patients had 10-100 cells, 20 patients had 100-300 ells and only 06 patients had numerous cells. P-value was found to be not significant (P-value >0.541). (Table-I).

**Table-I:** CSF cells count of study subjects: n = 166

*Age in months*	*CSF WBCs*			*Total*	*P value*
	*10-100 cell*	*100-300 cell*	*Numerous cell*		
01 - 24 months	19	97	22	138	0.541
25 - 59 months	02	20	06	28	
Total	21	117	28	166	

**Table-II:** Laboratory finding of LPA and PCR of study subject n = 166

*Result*	*LPA*	*PCR*	*P-VALUE*
Positive (+ve)	26	65	0.0001
Negative (-ve)	140	101	
TOTAL	166	166	

**Fig.1:**
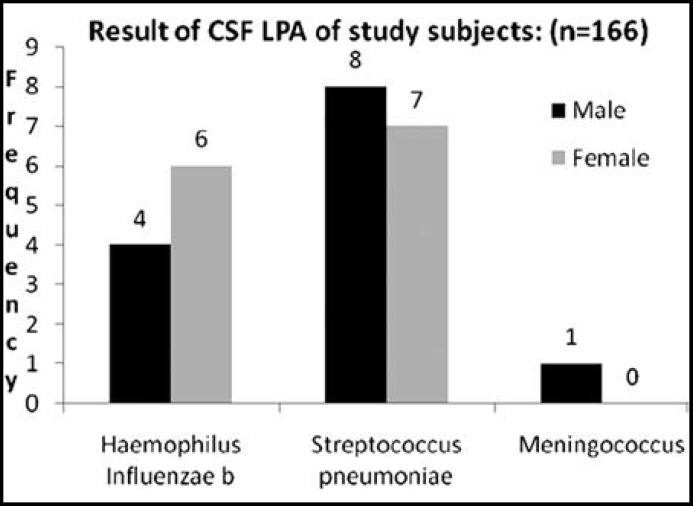
Result of CSF LPA of study subjects: (n=166)**.**

**Fig.2:**
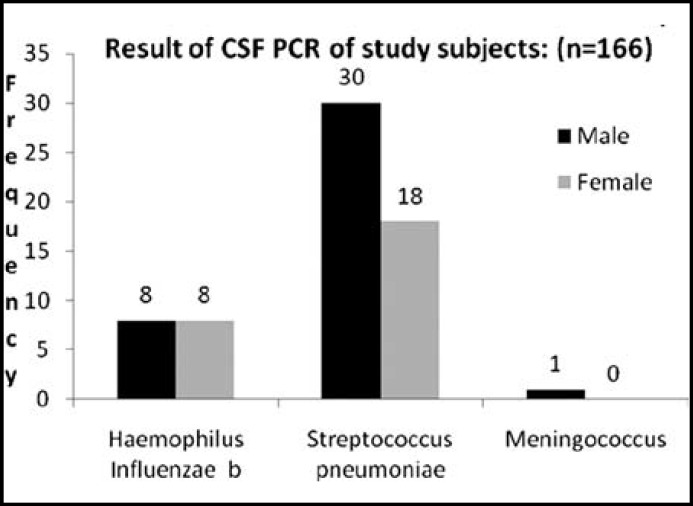
Result of CSF PCR of study subjects: (n=166).

The etiological agents identified by latex particle agglutination (LPA) were in 26/166 (15.66%) and the isolated organisms included Haemophilus influenzae type b (Hib) 10 cases, Strectococcus Pneumoniae 15 cases and meningococcus only 01 case. (Fig.1) PCR assays diagnosed bacterial meningitis in 65/166 (39.15%) cases. The organisms identified were Haemophilus influenzae type b, (Hib) 16 cases, S. Pneumoniae 48 cases and meningococcus only 1 case. (Fig.2) Among these 166 patients, LPA was positive in 26 patients and negative in 140 patients. PCR was found positive in 6 patients and were negative in 101 patients. P-value was calculated and was noted to be significant ( P-value <0.0001). (Table-II) Out of 166 patients, 9 (5.42%) patients expired, 7 left against medical advice (LAMA) and 150 patients improved and were discharged from the facility.

## DISCUSSION

Though LPA and PCR are very useful techniques in microbiology but unfortunately, very scanty data is available for PCR and LPA in bacterial meningitis in Pakistan.

Bacterial meningitis is one of the most common and life threatening infection of central nervous system (CNS) throughout the world. It is more common in developing countries. Rapid diagnosis and treatment is essential because of its grave complications like hearing loss, mental retardation, seizures and behavioral changes may occur in up to one half of the survivors.^3^

The gold standard for the diagnosis of bacterial meningitis is microscopic examination and culture of cerebrospinal fluid (CSF). However, this approach have some disadvantages with regard to desired rapidity and sensitivity.^13^ Proper treatment is guided by the result of culture which may take 24-48 hours to obtain; antibiotic susceptibility testing may need an additional 24 hours.^13^^,^^14^ Therefore, LPA and PCR techniques have been introduced for rapid identification of bacterial antigens. LPA can detect comparatively very small quantity of antigen present.

In the current study, the etiological agents identified by LPA were in 15.66% patients and the pathogens isolated were Haemophilus influenzae type b, Streptococcus Pneumoniae and meningococcus. A study by Surinder et al in India quoted 15.4% cases by LPA in his study which is quite compatible to our study.^15^ Similar studies conducted by neighboring countries like Iran, Bangladesh and Turkey identified bacterial antigens by LPA in 5.05%, 40% and 23% cases respectively.^16^^-^^18^ Although bacterial culture is considered to be the standard method, the negative effect of prior antibiotic use on its sensitivity necessitates use of non culture techniques for diagnosis. LPA and PCR may be employed for the diagnosis of bacterial meningitis in cases in which prior antibiotic use may inhibit CSF culture or growth.^19^

PCR is the most accurate and reliable method especially among patients with a history of antibiotic use before spinal tap.^20^ In our study PCR analysis was the more sensitive method confirming 65 cases (39.5%) among 166 patients. A similar study of PCR technique conducted by Cehan M et al quoted 59.6% patients by PCR technique.^18^ Ghotaslou et al picked 19 cases (6.85%) by PCR which is in contrast to our study.^16^

Although CSF culture is the diagnostic reference standard for bacterial meningitis, its sensitivity is limited particularly when antibiotic were previously administered. However, PCR is theoretically less affected by antibiotic. Henery M et al study showed that PCR was positive in 25.1% cases (51 S. Pneumoniae, 57 N. meningitidis and 5 H. influenzae).^21^ Administration of antibiotic prior to lumber puncture is a common situation leading to decreased culture yield.^22^

Therefore, PCR has been suggested as a rapid diagnostic test for bacterial meningitis. ^22^^,^^23^ Amplification of DNA from non-viable bacteria could potentially facilitate diagnosis in culture negative cases.

Nucleic acid amplification such as PCR do not require viable bacteria for a positive assay and generally considered to be highly sensitive.^24^

 A study from India by Das B.K showed that by using LPA test, an etiological diagnosis was made in 83% cases of bacterial meningitis. The sensitivity and specificity of LPA test was 83% and 100% respectively.^9^

LPA test was positive in 35.2% cases in a study by Ouedraogo SM et al.^26^ This variation in the result of these tests may be due the type and quality of kits used, various characteristics of patients and the collection and its transportation of sample to the laboratory facility.

## CONCLUSION

LPA and PCR were found to be useful technique for identification of bacterial antigen of H. Influenzae type b, S. pneumoniae and meningococcus in CSF samples of suspected meningitis. PCR is a rapid, sensitive and specific diagnostic test for bacterial meningitis. LPA also provided positive results especially when culture is negative and in patients who have received prior antibiotics.

## Recommendation:

- Sensitive diagnostic tests like LPA and PCR substantially improve identification of the three common pathogens causing meningitis like H. Influenzae S. Pneumonae and meningococcus.
- LPA and PCR should be routinely done when Gram stain is positive and abnormal CSF

values (WBC count, Glucose, Protein) indicate bacterial meningitis.

- LPA has its limitation as it cannot provide serotype information, whereas PCR can, therefore both are advised.
- Further local studies are needed to establish the true burden of these three common

bacterial pathogen causing meningitis in children.
